# Cystic Dilation of the Aqueductus Sylvii in Case of Trisomy 17p11.2—pter with the Deletion of the Terminal Portion of the Chromosome 6

**DOI:** 10.1155/2010/354170

**Published:** 2011-01-16

**Authors:** Emese Horváth, János Sikovanyecz, Attila Pál, László Kaiser, Bálint L. Bálint, Póliska Szilárd, Zoltán Kozinszky, János Szabó

**Affiliations:** ^1^Department of Medical Genetics, Faculty of Medicine, Albert Szent-Györgyi Medical and Pharmaceutical Centre, University of Szeged, Somogyi u. 4 H: 6720, 67256 Szeged, Hungary; ^2^Department of Obstetrics and Gynaecology, Faculty of Medicine, Albert Szent-Györgyi Medical and Pharmaceutical Centre, University of Szeged, Semmelweis u. 1, 67256 Szeged, Hungary; ^3^Department of Pathology, Faculty of Medicine, Albert Szent-Györgyi Medical and Pharmaceutical Centre, University of Szeged, Semmelweis u. 1, 67256 Szeged, Hungary; ^4^Department of Biochemistry and Molecular Biology, Clinical Genomics Centre, Medical and Health Science Centre, University of Debrecen, 4012 Debrecen, Hungary; ^5^Women and Children's Division, Department of Obstetrics and Gynecology, Oslo University Hospital, Ullevaal, 0407 Oslo, Norway

## Abstract

Since the 1970s, about 30 cases of partial or complete trisomy 17p have been presented in the literature. Partial trisomies of the short arm of chromosome 17 are somewhat more common, but complete trisomy is quite rare. Most of these cases were described in infants and newborns; and to our knowledge only 3 cases of trisomy 17p have been detected intrauterine. Phenotypic features of trisomy 17p in fetuses are intrauterine growth retardation, ventriculomegaly, cleft lip and cleft palate, micrognathia, horseshoe kidneys, single umbilical artery, and congenital heart defects. The sonographic and foetopathologic findings of a pregnancy trisomy 17p11.2—pter with the deletion of the terminal portion of the chromosome 6 due to paternal balanced translocation are described in this case report.

## 1. Introduction

Since the 1970s, about 30 cases of partial or complete trisomy 17p have been presented in the literature [1–5]. Partial trisomies of the short arm of chromosome 17 are somewhat more common, but complete trisomy is quite rare [1]. Most of these cases were described in infants and newborns, and to our knowledge only 3 cases of trisomy 17p have been detected intrauterine [6, 7]. Phenotypic features of trisomy 17p in fetuses are intrauterine growth retardation, ventriculomegaly, cleft lip and cleft palate, micrognathia, horseshoe kidneys, single umbilical artery, and congenital heart defects [6, 7]. 

The sonographic and foetopathologic findings of a pregnancy trisomy 17p11.2—pter with the deletion of the terminal portion of the chromosome 6 due to paternal balanced translocation are described in this case report.

## 2. Case Report

**Figure 1:**
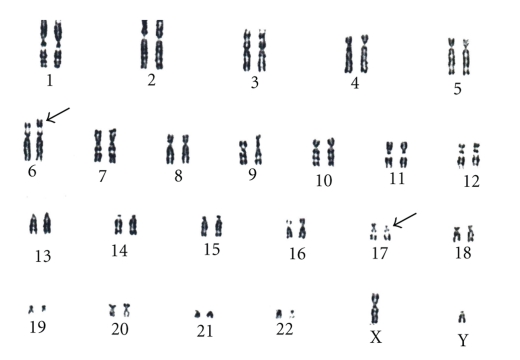
Karyotype of the father.

**Figure 2:**
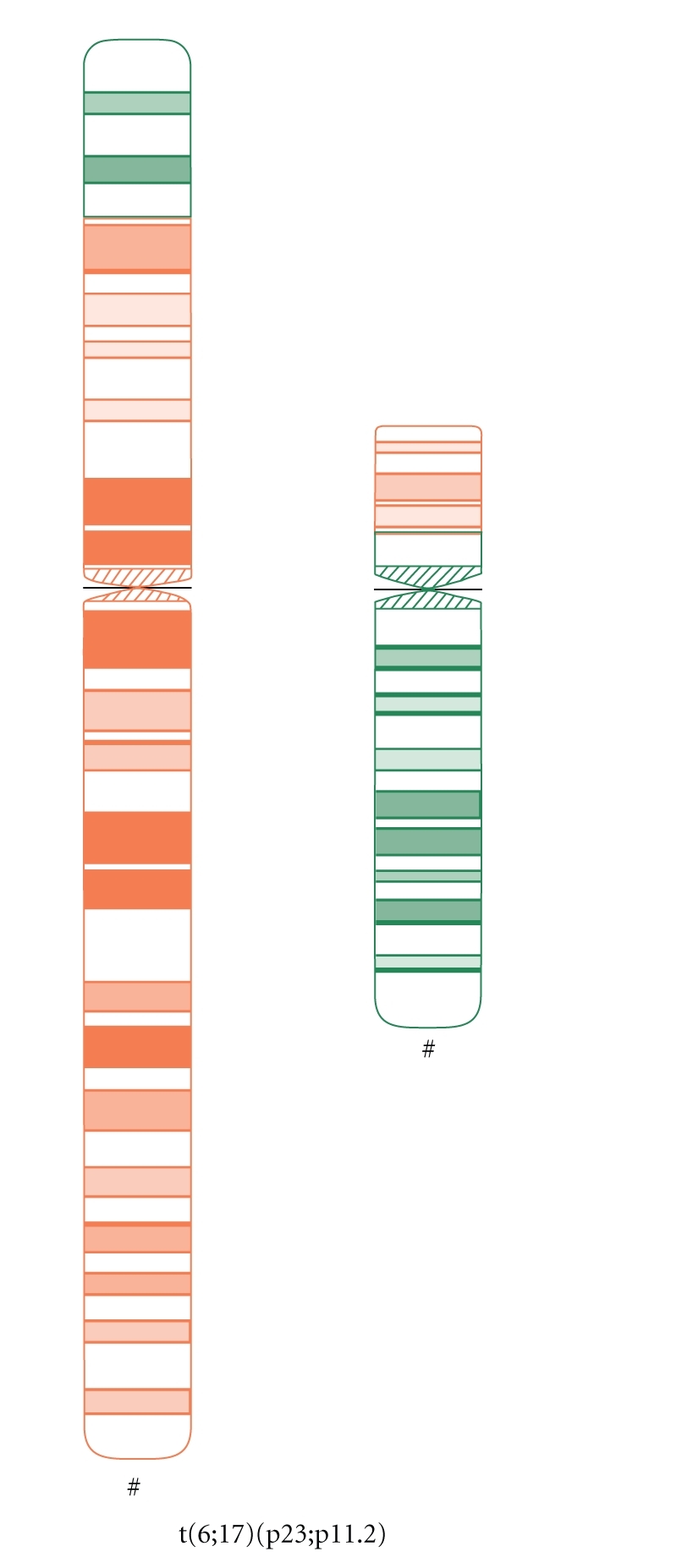
Idogram of the father.

An anxious (G2; P0) couple requested maternal and paternal chromosomal examination after a spontaneous abortion. At the time of the examination, the wife was 34 yrs, and the husband was 36 yrs old. Standard cytogenetic analysis on GTG-banded (at the 550 banding stage) chromosomes of the lymphocytes revealed 46,XX karyotype in the woman, and a balanced translocation 46,XY,t(6;17)(p25;p11.2) in the husband (Figure 1). Fluorescens in situ hybridisation (FISH) analysis by using probes specific for the subtelomeric regions of the short arm of chromosome 6 and chromosome 17 (17p13.3 and 6p) performed by Márta Czakó et al. in the Institut of Medical Genetics of University of Pécs (Hungary) modified the result of the G-banding cytogenetic analysis, which confirmed a balanced translocation with the result 46,XY,t(6;17)(p23;p11.2) (Figure 2).

**Figure 3:**
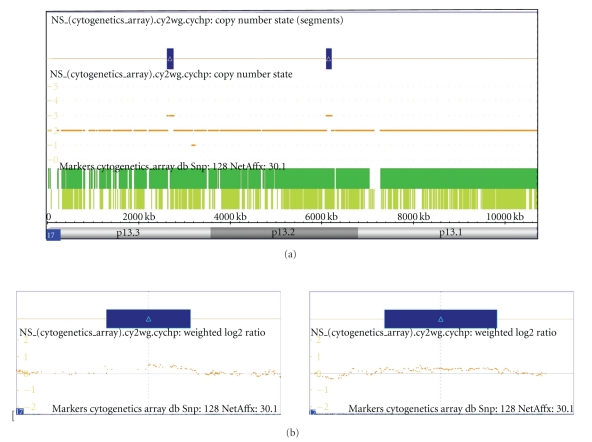
(a) Overview of the 17p13 region in the Cytogenetic array investigation. The two highlited regions show an incease from two to three copies in the genome. (b) Detailed view of the two regions with an increase in copy number. Dots represent the weighted log2 signal values for individual probes covering the inquired region.

Cytogenetic studies in the family members of the husband revealed the same translocation in his father and his sister. In order to refine the results of the cytogenetic test of the father, we performed an in-depth whole-genome cytogenetic microarray test using the Affymetrix Cytogenetic array that contains 2.7 million probes including SNP marker probes. We inquired the genome for gains and losses larger than 100 kbp with a minimum confidence of 85% and a minimum marker count of 35. The results are summarized in Table 1. Interestingly in the whole genome two regions were identified that were presenting increase in copy number both on chr 17 regions p13.2 and p13.3. Details of these two regions can be seen on Figure 3. Prenatal karyotyping was offered to the mother in the next pregnancy, because of the 10% risk of recurrence of the unbalanced fetal karyotype [8]. The first trimester sonographic scan was normal: the nuchal translucency thickness was 1.0 mm at 48 mm crown-rump length. After informed consent of the couple a standard cytogenetic analysis was performed on GTG-banded (at the 550 banding stage) chromosomes of cultured amniotic fluid with a Cytovision automatic imaging system. The foetal cytogenetic analysis demonstrated an unbalanced chromosomal rearrangement of paternal origin in all of the examined cells. The G-banding cytogenetic investigation showed a fetus with a trisomy 17 p11.2-pter. The FISH analysis by probes specific for the subtelomeric regions of the short arm of chromosome 6 and chromosome 17 confirmed that the paternal balanced translocation led to a trisomy 17 p11.2-pter with the deletion of the 6p25 in the fetus. The fetal karyotype was 46,XY,der(6)t(6,17)(p23;p11.2)pat. [9].

Performing a second trimester genetic sonogram in our department, the following fetal structural abnormalities were explored: severe mandibular hypoplasia, a hypoplastic nose and low-set ears, and moderate prefrontal subcutaneous oedema and abnormal “four-chamber” view: a ventricular septum defect, right ventricle hyperplasia and an echogenic focus in each ventricle, a single artery in the umbilical cord. The umbilical flow was in the normal range, and a sinus rhythm was detected. 

**Figure 4:**
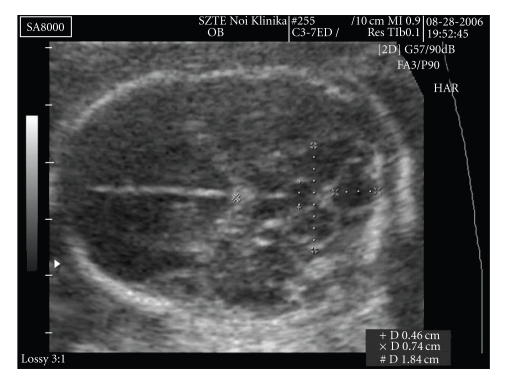
Cystic dilation of the aqueductus Sylvii.

The foetal brain structure was also abnormal. The 4th ventricle was dilated (AP diameter: 7.4 mm). On the anterior side of the cerebellum, behind the thalamus, there was a 4.6 × 5.0 mm cystic shadow (Figure 4).

The transversal diameter of the cerebellum was 19 mm (appropriate for week 19). The exact localization of the cystic shadow, whether it is in the cerebellar stroma or in front of it, could not be achieved ultrasonographically. 

After a fully informed consent, the couple requested termination of the pregnancy, which was performed by laminaria cervix dilatation and intramuscular Sulprostone (a prostaglandin 2F*α* derivate) administration. 

Fetopathological examination showed a hypoplastic mandible, low-set ears, a hypoplastic nose, a single umbilical artery, membranous ventricular septal defect, and truncus arteriosus communis, which were not described by the ultrasonographer. The shape of the base of the bony cranium was abnormal. The posterior fossa was narrowed. The cerebellum seemed slightly abnormal, but the transversal section demonstrated cystic dilation of the aqueductus Sylvii (Figure 4).

## 3. Discussion

We report the case of a prenatally detected unbalanced translocation of the short arm of chromosome 17, where the short arm over the p11.2 part was attached to the p23 region of chromosome 6.The translocation led to the deletion of the 6p25. The frequency of chromosome rearrangements in couples with reproductive failure is 3%–5% [8, 10]. Although parental cytogenetic study is indicated after 2 spontaneous abortions, the parents requested karyotype analysis, because of two previous pregnancies with unsuccessful outcome. A balanced translocation was found in the father: 46,XY,t(6;17)(p23; p11,2). The Affymetrix Cytogenetics Whole-Genome 2.7 M Array shows two insertions of minimum 100 kbp in the 17p13 region. One of this is affecting a coding gene: GARNL4 (GTPase activating Rap/RanGAP domain-like 4). It should be noted that these arrays are not able to localize the actual position of the duplicated region in the genome only the origin of the segments that have an increased copy number. Balanced translocation carriers may have chromosomally normal and abnormal foetuses; therefore, it is a classical indication for fetal karyotyping. A higher spontaneous abortion rate has been observed among chromosomally balanced carriers, due to the high selection rate of unbalanced gametes and the diminished viability of unbalanced zygotes. Earlier data pointed to a significantly higher spontaneous fetal loss rate in paternal than in maternal carriers [10]. On this basis, of the 10% a priori risk of an unbalanced fetal karyotype [8], a prenatal cytogenetic investigation was performed. The fetal cytogenetic analysis revealed an unbalanced fetal karyotype, trisomy of 17p11.2—pter with the deletion of the terminal portion of the chromosome 6 in all of the examined amniotic fluid cells. 

The phenotypical manifestation depends on the chromosomal breakpoints. The expected phenotypic features of trisomy 17p are microcephaly, mandibular hypoplasia, antimongoloid slanting of palpebral fissures, a high-arched palate, hypertelorism, low-set prominent ears, a short, webbed neck, hypotonia, small palpebral fissures, postnatal growth retardation, redundant neck skin folds, congenital heart defects, club foot, and severe mental retardation [1–5]. The 17p duplication includes the peripheral myelin protein 22 gene within the 17p12 band. This demonstrates a rather different phenotype, referred to as Charcot-Marie-Tooth disease type 1A with demyelinating neuropathy [11–13]. The manifestation of the duplication of the bands 17p11.2-p12 (prenatal and postnatal growth retardation, facial abnormalities, club foot, and mild developmental deficits) appears to be milder than other duplications of the short arm of chromosome 17 [14]. Cheng et al. investigated the differences in NT thickness among fetuses in which either parents is a balanced chromosome translocation carrier. A significantly greater NT thickness was shown in the unbalanced chromosomal translocation group compared with both the balanced chromosomal translocation group and the normal karyotype group. Two cases from this study involved chromosome 17. One of these cases, the nuchal translucency thickness was 3.1 mm, but in the other it was only 1.3 mm [15]. There is no doubt that first trimester ultrasound examination has a better detection rate in chromosomal abnormalities than that in the second trimester [16], but in our case the first trimester ultrasound scan was considered normal on the basis of the 1,0 mm nuchal translucency thickness at 48 mm crown-rump length. The second trimester ultrasound examination showed severe structural malformations, such as a hypoplastic nose, low-set ears, hypoplastic mandible, a single umbilical artery, heart defect, and brain malformation.

Most of these abnormalities had previously been described in prenatally diagnosed cases of partial or complete trisomy 17p [6, 7], except the cystic dilation of the aqueductus Sylvii associated with dilation of the 4th ventricle, and this is a new observation. The deletion of the terminal portion of the chromosome 6 could account for the differences found in this case [17]. Other abnormalities, such as intrauterine growth retardation and horseshoe kidney, were missing in our case.

It is known from the literature that the phenotypical manifestation of partial 17p trisomy and 6p25 deletion syndrome is clinically severe, which helped the parents to make an informed decision about the future of the pregnancy. The foetopathologic examination confirmed the prenatally detected abnormalities. A detailed foetopathological study may help to read phenotypic expression of the chromosomal rearrangement detected by cytogenetic and FISH investigations. A precise exploration of foetal abnormalities may assist in counselling with the couple in order to decide about the future prospect of pregnancy.

## Figures and Tables

**Table 1:** Results of the affymetrix cytogenetics whole-genome 2.7 M array.

Cytoband start	p11.2	q24.1	q22.1	p13.3	p13.2
Cytoband end	p11.2	q24.1	q22.1	p13.3	p13.2
CN state	0	1	1	3	3
Type	Loss	Loss	Loss	Gain	Gain
Chromosome	Y	2	6	17	17
Min	9235741	156914391	116263486	2653130	6117392
Max	9972947	157022143	116369551	2792389	6227251
Size (kbp)	737	107	106	139	109
Mean marker distance	6641	1584	1309	3978	1007
Marker count	112	69	82	36	110
Confidence %	87.8	85.6	85.3	92.1	87.2
FISH Clones		RP11-605B16	RP1-188H10, RP11-721G11	CTB-11O23	
Genes	TSPY3, TSPY1, CYorf16, TSPY3, CYorf16, CYorf16, CYorf16, CYorf16	GPD2, GPD2	FRK	GARNL4, GARNL4	

## References

[1] Mikhail FM, McIlvried D, Holt RL, Messiaen L, Descartes MD, Carroll AJ (2006). Complete trisomy 17p syndrome in a girl with der(14)t(14;17)(p11.2;p11.2). *American Journal of Medical Genetics*.

[2] Morelli SH, Deubler DA, Brothman LJ, Carey JC, Brothman AR (1999). Partial trisomy 17p detected by spectral karyotyping. *Clinical Genetics*.

[3] Rethore MO, Renault F, Lafourcade J (1983). 17p trisomy. *Semaine des Hopitaux*.

[4] Schrander-Stumpel C, Schrander J, Fryns JP, Hamers G (1990). Trisomy 17p due to a t(8;17) (p23;p11.2)pat translocation. Case report and review of the literature. *Clinical Genetics*.

[5] Shaffer LG, McCaskill C, Hersh JH, Greenberg F, Lupski JR (1996). A clinical and molecular study of mosaicism for trisomy 17. *Human Genetics*.

[6] De Pater JM, Van Tintelen JP, Stigter R, Brouwers HAA, Scheres JMJC (2000). Precarious acrocentric short arm in prenatal diagnosis: no chromosome 14 polymorphism, but trisomy 17p. *Genetic Counseling*.

[7] Kulharya AS, Garcia-Heras J, Radtke HB, Norris KS, Keppen LD, Flannery DB (1998). Prenatal diagnosis of a trisomy 17p derived from a de novo non-mosaic satellited marker. *Clinical Genetics*.

[8] Szabó J, Szörényi Á, Szemere G (1984). Unbalanced translocation: one of the genetic causes of recurrent abortions. *Orv Hetil*.

[9] Shaffer LG, Tommerup N (2005). *An international System for Human Cytogenetic Nomenclature*.

[10] Fortuny A, Carrio A, Soler A, Cararach J, Fuster J, Salami C (2006). Detection of balanced chromosome rearrangements in 445 couples with repeated abortion and cytogenetic prenatal testing in carriers. *Fertility and Sterility*.

[11] Chance PF, Bird TD, Matsunami N, Lensch MW, Brothman AR, Feldman GM (1992). Trisomy 17p associated with Charcot-Marie-Tooth neuropathy type 1A phenotype: evidence for gene dosage as a mechanism in CMT1A. *Neurology*.

[12] Roa BB, Greenberg F, Gunaratne P (1996). Duplication of the PMP22 gene in 17p partial trisomy patients with Charcot-marie-tooth type-1A neuropathy. *Human Genetics*.

[13] Vogt J, Hill S, Brueton L (2006). Partial trisomy 17p12pter, associated with pre andpostnatal growth retardation, dysmorphic facial anddigital features, developmental delay, andsigns ofHMSN1 inearly childhood. *European Journal of Medical Genetics*.

[14] Kozma C, Meck JM, Loomis KJ, Galindo HC (1991). De novo duplication of 17p [dup(17)(p12-p11.2)]: report of an additional case with confirmation of the cytogenetic, phenotypic, and developmental aspects. *American Journal of Medical Genetics*.

[15] Cheng POJ, Chang SD, Shaw SW, Soong YK (2005). Nuchal translucency thickness in fetuses with chromosomal translocation at 11-12 weeks of gestation. *Obstetrics and Gynecology*.

[16] Nicolaides KH (2004). *The 11-13+6 Weeks Scan*.

[17] Van Der Knaap MS, Kriek M, Overweg-Plandsoen WCG (2006). Cerebral white matter abnormalities in 6p25 deletion syndrome. *American Journal of Neuroradiology*.

